# Supplementary material for: Exploring global and local processes underlying alterations in resting-state functional connectivity and dynamics in schizophrenia
Source: Front Psychiatry. 2024 Feb 13;15:1352641. doi: 10.3389/fpsyt.2024.1352641 (PMC10897003; doi:10.3389/fpsyt.2024.1352641)
Supplement: Supplementary file 1 [file DataSheet_1.pdf]

Supplementary Material

1 SUPPLEMENTARY TABLES AND FIGURES

1.1 Figures

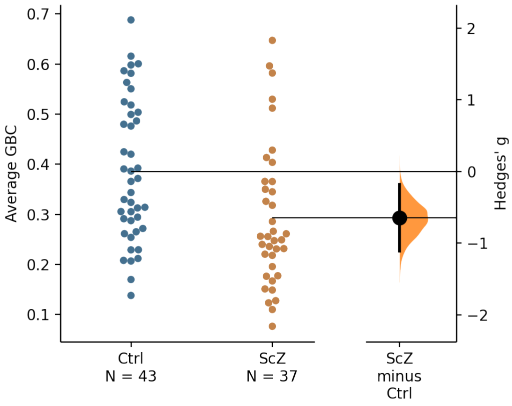

Figure 1a. Average global GBC across participants.

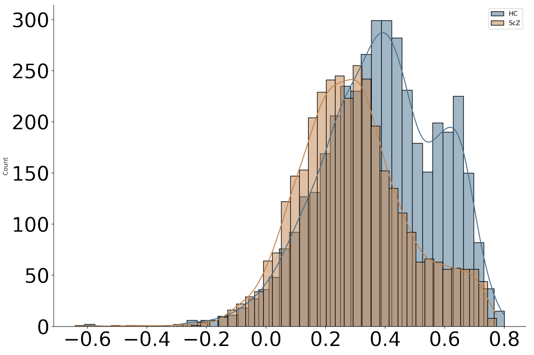

Figure 1b. Histogram of GBC across participants.

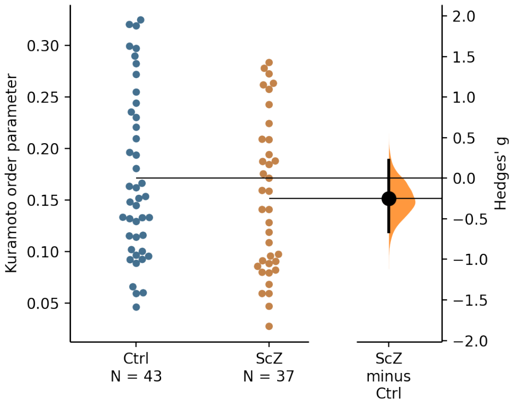

Figure 1c. Global synchrony (Kuramoto order parameter) across participants.

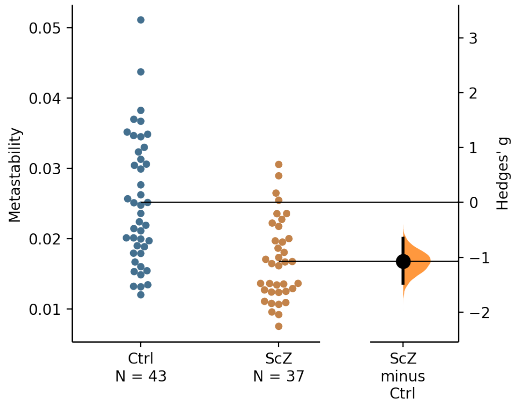

Figure 1d. Global metastability across participants.

Figure 1. Global differences in functional connectivity and temporal dynamics. a) Comparison of average GBC per participant for the two groups. Individual dots represent average GBC for one participant. The difference plot on the right shows the difference between the groups in terms of effect size. b) Histogram of region-wise GBC values for the two groups. The histogram displays the region-wise GBC data pooled for all participants in each group. c) Synchrony comparison between the two groups. Each dot represents the mean Kuramoto order parameter (a measure of synchrony) for one participant. The difference plot on the right shows the group difference in terms of effect size. d) Metastability comparison between the two groups. Each dot represents the metastability of one participant. The difference plot on the right shows the group difference in terms of effect size.

1.2 Tables

Region	Hedges' g	p value
Precentral L	-0.69	0.0024
Precentral R	-0.68	0.0024
Frontal Sup 2 L	-0.70	0.0018
Frontal Sup 2 R	-0.49	0.0318
Frontal Mid 2 L	-0.56	0.0126
Frontal Mid 2 R	-0.43	0.0608
Frontal Inf Oper L	-0.72	0.0014
Frontal Inf Oper R	-0.67	0.0042
Frontal Inf Tri L	-0.68	0.0030
Frontal Inf Tri R	-0.59	0.0092
Frontal Inf Orb 2 L	-0.53	0.0212
Frontal Inf Orb 2 R	-0.41	0.0644
Rolandic Oper L	-0.61	0.0078
Rolandic Oper R	-0.67	0.0032
Supp Motor Area L	-0.75	0.0006
Supp Motor Area R	-0.84	0.0006
Olfactory L	-0.26	0.2536
Olfactory R	-0.16	0.4704
Frontal Sup Medial L	-0.51	0.0242
Frontal Sup Medial R	-0.52	0.0206
Frontal Med Orb L	-0.40	0.0736
Frontal Med Orb R	-0.42	0.0600
Rectus L	-0.52	0.0264
Rectus R	-0.32	0.1518
OFCmed L	-0.47	0.0404
OFCmed R	-0.31	0.1728
OFCant L	-0.30	0.1932
OFCant R	-0.28	0.2026
OFCpost L	-0.43	0.0578
OFCpost R	-0.51	0.0186
OFClat L	-0.60	0.0094
OFClat R	-0.13	0.5442
Insula L	-0.74	0.0018
Insula R	-0.65	0.0050
Cingulate Ant L	-0.49	0.0296
Cingulate Ant R	-0.51	0.0248
Cingulate Mid L	-0.87	0.0002
Cingulate Mid R	-0.82	0.0002
Cingulate Post L	-0.57	0.0132
Cingulate Post R	-0.35	0.1174
Calcarine L	-0.82	0.0002
Calcarine R	-0.76	0.0016
Cuneus L	-0.77	0.0010
Cuneus R	-0.61	0.0064
Lingual L	-0.89	<0.0001
Lingual R	-0.85	0.0002

Table S1. Regional effect sizes Effect sizes and p values for the comparison of the control and the patient group in average GBC for each of the 90 regions of the AAL2 parcellation.

Region	Hedges' g	p value
Occipital Sup L	-0.71	0.0012
Occipital Sup R	-0.72	0.0024
Occipital Mid L	-0.85	0.0002
Occipital Mid R	-0.92	<0.0001
Occipital Inf L	-0.84	0.0004
Occipital Inf R	-0.56	0.014
Fusiform L	-0.89	0.0002
Fusiform R	-0.98	<0.0001
Postcentral L	-0.70	0.0024
Postcentral R	-0.70	0.002
Parietal Sup L	-0.85	<0.0001
Parietal Sup R	-0.94	<0.0001
Parietal Inf L	-0.85	<0.0001
Parietal Inf R	-0.51	0.025
SupraMarginal L	-0.56	0.0132
SupraMarginal R	-0.61	0.0072
Angular L	-0.53	0.0184
Angular R	-0.51	0.0234
Precuneus L	-0.88	0.003
Precuneus R	-0.69	0.001
Paracentral Lobule L	-0.72	0.0126
Paracentral Lobule R	-0.58	0.0106
Heschl L	0.59	0.0028
Heschl R	-0.70	<0.0001
Temporal Sup L	-0.96	<0.0001
Temporal Sup R	-0.83	0.0076
Temporal Pole Sup L	-0.6	0.001
Temporal Pole Sup R	-0.77	0.001
Temporal Mid L	-0.81	0.0008
Temporal Mid R	-0.87	<0.0001
Temporal Pole Mid L	-0.68	0.0026
Temporal Pole Mid R	-0.77	0.0008
Temporal Inf L	-0.37	0.0972
Temporal Inf R	-0.6	0.009

Table S2. Regional effect sizes - ctd. Effect sizes and p values for the comparison of the control and the patient group in average GBC for each of the 90 regions of the AAL2 parcellation.

	Mean difference	Hedges' g	95% CI	p value
Global cortical GBC Global	-0.09	-0.65	[-1.11 -0.14]	p=0.0080
cortical synchrony Global	-0.10	-0.34	[-0.79 0.19]	p=0.0626
cortical metastability	-0.006	-0.40	[-0.84 0.08]	p=0.0946
GBC Sensory areas	-0.12	-0.77	[-1.25 -0.26]	p=0.0026
GBC Association areas	-0.09	-0.59	[-1.05 -0.08]	p=0.0134
GBC Somato-motor (SomMot)	-0.12	-0.4	[-1.22 -0.23]	p=0.0036
GBC Control (Cont)	-0.09	-0.57	[-1.05 -0.07]	p=0.0188
GBC Default mode (Def)	-0.08	-0.55	[-1.02 -0.04]	p=0.0250
GBC Salience/Ventral attention (Sal/VAttn)	-0.10	-0.66	[-1.12 -0.16]	p=0.0062
GBC Dorsal attention (DAttn)	-0.12	-0.82	[-1.30 -0.28]	p=0.0014
GBC Limbic (Lim)	-0.08	-0.63	[-1.07 -0.14]	p=0.0104
GBC Visual (Vis)	-0.11	-0.74	[-1.23 -0.23]	p=0.0030

Table S3. Local and global group differences. Overview of the global and local differences in functional connectivity and temporal dynamics between the healthy control and the ScZ patient group. In this analysis, as opposed to the main manuscript, a stricter exclusion threshold for framewise displacement of 0.3 was, resulting in the exclusion of 4 control participants and 5 schizophrenia patients.

	95%	90%	85%	80%
Avg. global GBC	0.001 [0.02]	0.003 [0.04]	0.003 [0.04]	0.003 [0.04]
Avg. GBC sen.	0.002 [0.02]	0.003 [0.04]	0.003 [0.03]	0.002 [0.02]
Avg. GBC ass.	0.001 [0.02]	0.004 [0.04]	0.005 [0.06]	0.006 [0.06]
Synchrony	0.003 [0.04]	0.006 [0.08]	0.008 [0.10]	0.006 [0.08]
Metastability	-0.001 [-0.04]	-0.002 [-0.05]	-0.002 [-0.05]	-0.001 [-0.02]
	75%	70%	65%	60%
Avg. global GBC	0.004 [0.05]	0.007 [0.08]	0.005 [0.06]	0.013 [0.16]
Avg. GBC sen.	0.003 [0.03]	0.005 [0.06]	0.003 [0.03]	0.012 [0.13]
Avg. GBC ass.	0.007 [0.08]	0.010 [0.11]	0.008 [0.09]	0.016 [0.19]
Synchrony	0.009 [0.12]	0.001 [0.01]	-0.003 [-0.04]	-0.005 [-0.06]
Metastability	-0.005 [-0.14]	-0.001 [-0.02]	0.004 [0.13]	0.007 [0.22]

Table S4. ScZ-associated changes of GABA parameters Comparison of average global GBC, average GBC in sensory areas, average GBC in association areas, average synchrony and average metastability for different conditions with reduced GABAergic output from 95% to 60% in steps of 5%. Shown are the mean differences (i.e. the mean of the default condition minus the respective reduced GABA condition. The mean in each condition is calculated over the 40 virtual subjects.) and in brackets the effect size (Hedge's g). Significant differences, i.e. a permutation p value of < 0.001 is highlighted in bold. Permutation tests were performed using 5,000 permutations of labels.

	95%	90%	85%	80%
Avg. global GBC	0.008 [0.09]	0.006 [0.07]	0.003 [0.04]	0.010 [0.11]
Avg. GBC sen.	0.005 [0.07]	0.004 [0.05]	0.001 [0.07]	0.005 [0.06]
Avg. GBC ass.	0.010 [0.12]	0.007 [0.08]	0.005 [0.06]	0.010 [0.12]
Synchrony	0.011 [0.14]	-0.004 [-0.06]	-0.005 [-0.07]	-0.005 [-0.06]
Metastability	-0.006 [-0.17]	0.005 [0.14]	0.006 [0.18]	0.004 [0.13]
	75%	70%	65%	60%
Avg. global GBC	-0.003 [-0.03]	-0.002 [-0.02]	0.012 [0.15]	0.006 [0.08]
Avg. GBC sen.	-0.008 [-0.09]	-0.010 [-0.11]	0.005 [0.06]	-0.005 [-0.06]
Avg. GBC ass.	-0.003 [-0.03]	-0.005 [-0.05]	0.007 [0.08]	-0.001 [-0.01]
Synchrony	-0.013 [-0.16]	-0.013 [-0.15]	-0.017 [-0.20]	-0.024 [-0.31]
Metastability	0.003 [0.09]	0.004 [0.12]	0.001 [0.03]	0.010 [0.29]

Table S5. ScZ-associated changes of glutamatergic parameters Comparison of average global GBC, average GBC in sensory areas, average GBC in association areas, average synchrony and average metastability for different conditions with reduced glutamatergic output from 95% to 60% in steps of 5%. Shown are the mean differences (i.e. the mean of the default condition minus the respective reduced glutamate condition. The mean in each condition is calculated over the 40 virtual subjects.) and in brackets the effect size (Hedge's g). Significant differences, i.e. a permutation p value of < 0.001 is highlighted in bold. Permutation tests were performed using 5,000 permutations of labels.

	Hedges' g	Achieved power
Global cortical GBC Global	-0.65	0.82
cortical synchrony Global	-0.44	0.50
cortical metastability	-0.39	0.41
GBC Sensory areas	-0.78	0.93
GBC Association areas	-0.61	0.77
GBC Somato-motor (SomMot)	-0.74	0.91
GBC Control (Cont)	-0.57	0.72
GBC Default mode (Def)	-0.57	0.72
GBC Salience/Ventral attention (Sal/VAttn)	-0.69	0.86
GBC Dorsal attention (DAttn)	-0.83	0.96
GBC Limbic (Lim)	-0.59	0.74
GBC Visual (Vis)	-0.77	0.93

Table S6. Post-hoc analysis of achieved power. Overview of the achieved power for the group comparisons presented in Table 3 of the main manuscript. Achieved power was calculated based on sample and effect sizes (Hedge's g) using GPower 3.1 (Faul et al. (2007, 2009)).

REFERENCES

Faul, F., Erdfelder, E., Buchner, A., and Lang, A.-G. (2009). Statistical power analyses using g* power 3.1: Tests for correlation and regression analyses. *Behavior research methods* 41, 1149–1160

Faul, F., Erdfelder, E., Lang, A.-G., and Buchner, A. (2007). G* power 3: A flexible statistical power analysis program for the social, behavioral, and biomedical sciences. *Behavior research methods* 39, 175–191
